# Supplementary figures and images for: Was the Devonian placoderm Titanichthys a suspension feeder?
Source: R Soc Open Sci. 2020 May 20;7(5):200272. doi: 10.1098/rsos.200272 (PMC7277245; doi:10.1098/rsos.200272)

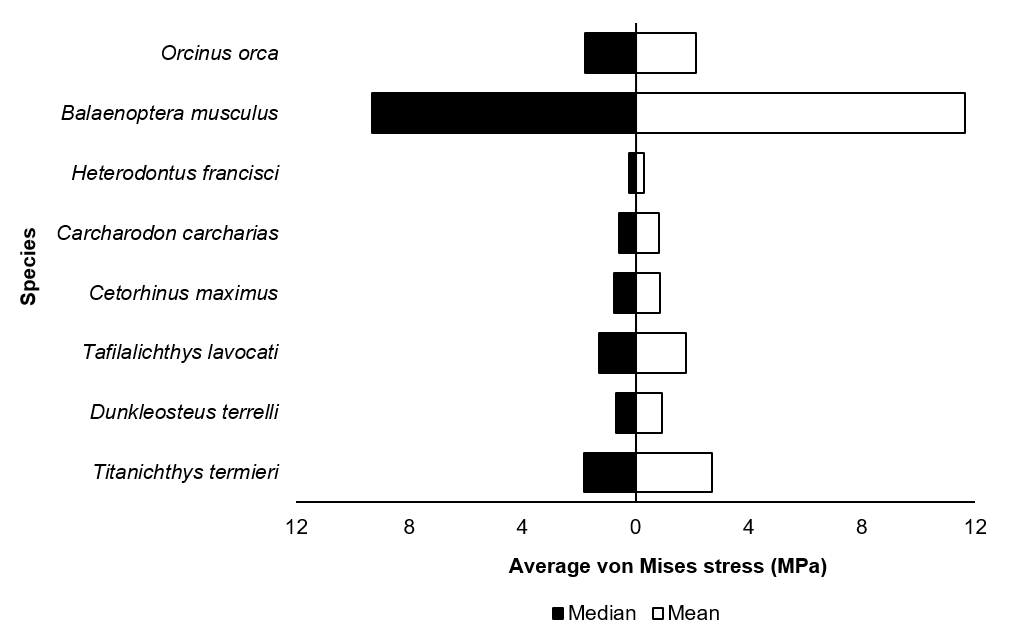

Supplement: Supplementary Figure 1 [file rsos200272supp1.tif]

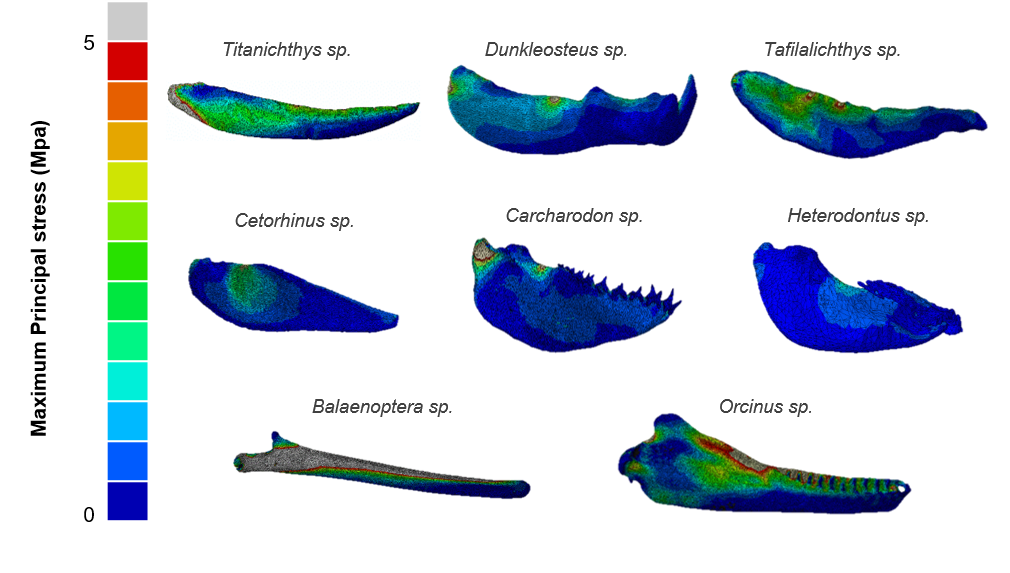

Supplement: Supplementary Figure 2 [file rsos200272supp2.tif]

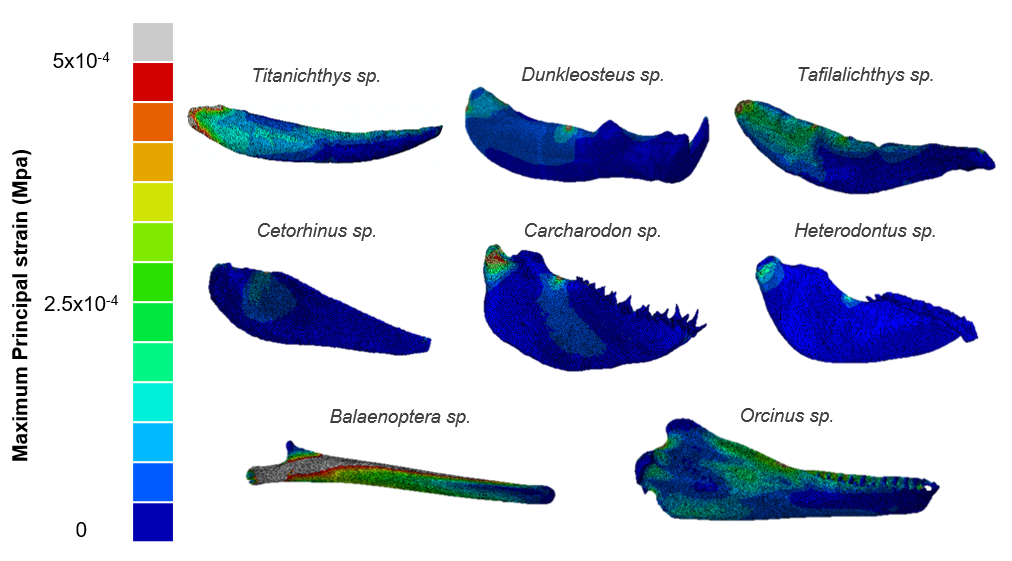

Supplement: Supplementary Figure 3 [file rsos200272supp3.tif]
